# Editorial: Polydopamine-based structures innovation for surface engineering and Musculoskeletal Tissue Regeneration

**DOI:** 10.3389/fbioe.2023.1153663

**Published:** 2023-02-10

**Authors:** Farnaz Ghorbani, Behafarid Ghalandari, Aldo R. Boccaccini, Derek H. Rosenzweig

**Affiliations:** ^1^ Department of Materials Science and Engineering, Institute of Biomaterials, University of Erlangen-Nuremberg, Erlangen, Germany; ^2^ Institute of Orthopaedic and Musculoskeletal Science, Royal National Orthopaedic Hospital, University College London, London, United Kingdom; ^3^ State Key Laboratory of Oncogenes and Related Genes, Institute for Personalized Medicine, School of Biomedical Engineering, Shanghai Jiao Tong University, Shanghai, China; ^4^ Department of Surgery, The Research Institute of McGill University Health Centre, McGill University, Montreal, QC, Canada

**Keywords:** podydopamine, surface engineering, musculoskeletal, tissue engineering, coating

Musculoskeletal disorders (MSDs) lead to a costly challenge for patients. The conditions range from temporary impairments to those that result in permanent disability and functional limitations (Musculoskeletal health, 2022). According to Global Burden of Disease 2019 data, approximately 1.71 billion people in the world suffer from MSDs, such as back/neck pain, fractures, osteoarthritis, and rheumatoid arthritis (Cieza et al., 2020). People with MSDs generally have limited mobility and reduced functioning, which restricts their ability to integrate into society. The regeneration of MSD following surgical interventions or prostheses has been problematic due to size dependency, fibrocartilage tissue formation, and poor biomechanical restoration (Glyn-Jones et al., 2015; Liu et al., 2019). It is therefore becoming increasingly promising to use tissue and surface engineering strategies to treat MSDs, using bio-inspired polymers as building blocks (Berthiaume and Yarmush, 2003). However, translation of research findings into practice still remains a challenge.

Bioinspired polymers are an advantageous choice when it comes to the development of MSD regeneration. Over the last few decades, it has been discovered that dopamine, a hormone and neurotransmitter, can also be used in polymeric form (under spontaneous oxidative polymerization) in tissue and surface engineering applications (Ghalandari et al., 2021). Polydopamine (PDA) effectiveness has expanded its application in bioengineering as nanoparticles and carriers. This melanin-like material is also used for regenerating bone, cartilage, muscle, nerves, and tendons due to its bioactivity, hydrophilicity, bioadhesion, and thermal stability. PDA’s success in tissue engineering field is attributed to its capacity to regulate tissue and cellular responses, including cell adhesion, proliferation, and to initiate repair and immune responses.

It has become increasingly popular to modify the surface of biomaterials by using PDA containing highly reactive functional groups that adhere to nearly all substrates. However, coating considerations, including monomer concentration, temperature, pH, and coating time, should be taken into account, affecting the homogeneity and uniformity of the coating layer. Orthopaedic surgeons often encounter difficulty achieving satisfactory osseointegration in treating MSD patients with conventional implants. Therefore, PDA is capable of modifying implant surfaces, increasing the adsorption of external components, thereby forming PDA-based multilayered coatings that are capable of improving bonding between synthetic constructs and bone surfaces, inducing osteogenic properties, and enhancing new bone formation by combining immunomodulation, angiogenesis, antibiosis, and antitumor properties. In a collected paper in this Research Topic, the application of catecholic amino acid 3,4-dihydroxyphenylalanine (DOPA) surface coating chelated with vascular endothelial growth factor has been reported by Huang et al. on allogeneic bone. A rabbit bone defect model was used to evaluate the ability of bone to regenerate after 12 weeks. Allogeneic bone bonded with DOPA has been found to have a similar surface microenvironment to the natural allogeneic bone so that the bone cortex was continuous and intact. A similar study in this Research Topic was conducted by Ghorbani et al. in which alginate dialdehyde-gelatin scaffolds were modified with PDA for bone regeneration. PDA-decorated matrices greater mechanical stability, durability and bioactivity potential than PDA-free matrices. MG-63 cell adhesion, viability, and proliferation were enhanced by PDA-coated scaffolds. Besides enhancing alkaline phosphatase secretion, mineralization intensity also increased, suggesting that the scaffolds may be capable of promoting bone regeneration.

Although PDA coating can improve cell adhesion and differentiation, a significant knowledge gap remains related to PDA-protein interactions. Recent studies have focused on understanding the interaction between PDA and proteins in the regeneration of tissues. A collected paper in this Research Topic used theoretical energy analysis and demonstrated that PDA-functionalized alginate dialdehyde-gelatin scaffolds interact spontaneously with osteomodulin and osteocalcin, resulting in the formation of a protein layer on the surface, suggesting potential applications in bone regeneration. Similar collected paper presented by Harati et al. demonstrated that cell adhesion and migration were affected by PDA decoration on gelatin-coated surfaces, arising from extracellular matrix proteins’ bioactivity as a function of PDA modification. Here, human adipose-derived stem cells reduced focal adhesion by downregulating the expression of integrins such as αV, α1, α2, and β1 on the PDA-gelatin compared to the PDA-free substrate.

Briefly, biocompatibility, hydrophilicity, and adhesion properties of light-responsive PDA as well as high bioactivity and osteogenicity are expected to expand its application in tissue/surface engineering for treatment of MSDs.

This Research Topic in Frontiers Bioengineering and Biotechnology-Biomaterials section has attracted eight papers (research/review) from researchers worldwide working on polydopamine-containing constructs, and it is expected to present a pathway for translation of research outputs to clinical practice.
